# Intraoperative detection of the remnant cystic duct during robot-assisted surgery using near-infrared fluorescence imaging: a case report

**DOI:** 10.1186/s12893-019-0567-8

**Published:** 2019-08-07

**Authors:** Labrinus van Manen, Quirijn R. J. G. Tummers, Akin Inderson, Abha Bhalla, Alexander L. Vahrmeijer, Bert A. Bonsing, J. Sven. D. Mieog

**Affiliations:** 1 Department of Surgery, Leiden University Medical Center, Albinusdreef 2, 2300 RC Leiden, The Netherlands; 2 Department of Gastroenterology, Leiden University Medical Center, Leiden, The Netherlands; 3 Department of Gastroenterology, Haga Hospital, The Hague, The Netherlands

**Keywords:** Robot-assisted surgery, Bile duct, Near-infrared fluorescence, Indocyanine green, Post cholecystectomy syndrome, Case report

## Abstract

**Background:**

Post cholecystectomy syndrome is characterized as recurrence of symptoms as experienced before cholecystectomy. In rare cases, a remnant cystic duct is causing these symptoms and occasionally surgical resection is performed. During surgery, visualization of the biliary ducts could be difficult due to inflammation and dense adhesions.

**Case presentation:**

In this article, we presented a 36-year old woman with post-cholecystectomy syndrome in which we evaluated the feasibility of near-infrared (NIR) fluorescence imaging using indocyanine green (ICG) for visualization of the remnant cystic and common bile duct during robot-assisted surgery. Intraoperative visualization of the remnant biliary duct and other important structures was feasible, and resection of the remnant cystic duct was successfully performed under fluorescence guidance, without any complications.

**Conclusions:**

NIR fluorescence imaging of the biliary ducts using ICG does not prolong the operating time, and could potentially decrease the operation time in difficult procedures, because of easy and fast detection of the biliary tract. Furthermore, it is a non-hazardous and non-invasive technique, as it does not require use of radiation and cannot cause bile duct injury. This case illustrated that ICG NIR fluorescence imaging during difficult robot-assisted surgical procedures of the bile ducts is effective and therefore highly recommended.

**Electronic supplementary material:**

The online version of this article (10.1186/s12893-019-0567-8) contains supplementary material, which is available to authorized users.

## Background

Cholecystectomy is the most common surgical procedure for patients with symptomatic cholecystolithiasis. Currently, minimally invasive surgery, either laparoscopic or robot-assisted, is the standard approach. Unfortunately, in some patients symptoms, such as abdominal pain and dyspepsia might persist after surgery, which has been defined as the post cholecystectomy syndrome (PCS) [1]. A remnant cystic duct, defined as a residual duct longer than 1 cm, could be one of the causes of PCS, although the true incidence is uncertain [2]. Treatment depends on the severity of symptoms and a resection of the remnant cystic duct is occasionally performed. Especially during laparoscopic or robot-assisted surgery, inflammation and dense adhesions hamper good visualization of the remnant cystic duct. This could result in serious complications, such as bile duct injury (BDI) [3, 4]. Today, no well-established, non-invasive and non-hazardous imaging techniques are available to guide the surgeon intraoperatively during such interventions. Intraoperative radiographic cholangiography (IOC) is sometimes used for bile duct visualization. However, especially in case of post cholecystectomy syndrome, it is difficult to navigate and to cannulate the common bile duct (CBD). Near-infrared (NIR) fluorescence imaging, is a new technique that has recently been evaluated during open and minimally invasive surgery [5]. In general, a fluorophore is excited and detected by specialized NIR fluorescence camera systems, in order to visualize the tissue of interest. The fluorophore indocyanine green (ICG), which is rapidly cleared by the liver after intravenous (IV) injection, has been extensively used for visualization of the bile ducts and many other applications [6]. In this case report, we describe the feasibility of NIR fluorescence imaging using ICG for visualization of the remnant cystic duct during robot-assisted surgery. This case report was described according to the SCARE guidelines [7].

## Case presentation

A 36-year old woman presented with an acute biliary pancreatitis at a peripheral hospital, after which a laparoscopic cholecystectomy was performed. One year later, she developed several episodes of acute onsets of abdominal pain, which were caused by biliary pancreatitis. Endoscopic ultrasound (EUS) revealed a high suspicion on presence of small bile stones in the remnant cystic duct. Although, Magnetic resonance cholangiopancreaticography (MRCP) did not show either an evident remnant biliary duct or presence of bile stones in either the remnant cystic duct or CBD, which could be explained by the limited image quality due to artefacts. Moreover, it has been shown that MRCP has a 93% sensitivity for detection of bile stones [8]. The a priori probability on presence of CBD stones was assessed as low, as no biochemical evidence of post hepatic obstruction (elevated bilirubin) was present. Based on this, patient was referred to an university hospital to investigate the possibility to remove the bile stones in the remnant cystic duct either endoscopically or surgically., Given the clinical symptoms, the findings at EUS (length of remnant cystic duct and suspicion on small bile stones in remnant cystic duct) and MRCP, the patient opted for a definitive therapeutic approach (surgical resection of remnant cystic duct) instead of another diagnostic modality (endoscopic retrograde cholangio-pancreatography), although this could have had therapeutic implications. Consequently, the case was discussed at the hepatopancreaticobiliary (HPB) multidisciplinary team meeting and a robot-assisted surgical resection of the remnant cystic duct under NIR fluorescence guidance using ICG was proposed. After screening for contraindications for the use of ICG (impaired kidney function, hypo/hyperthyroidism, pregnancy, and allergy for iodine), the patients provided informed consent for the use of ICG NIR fluorescence imaging.

**Fig. 1:**
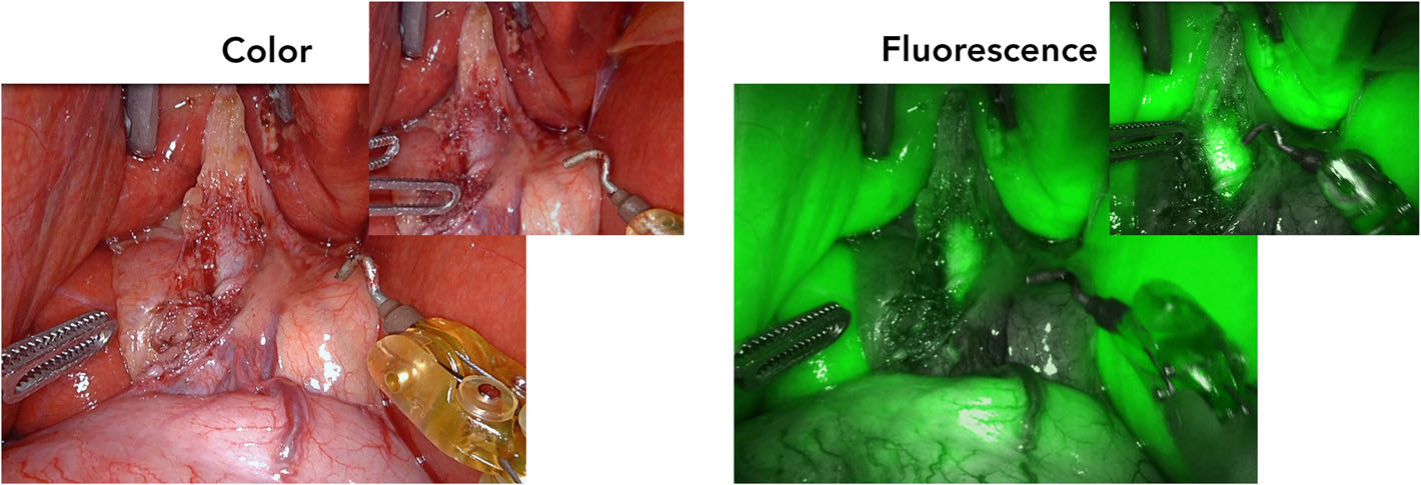
Representative color and fluorescence image with an overview of the liver hilum after some adhesiolysis. Insets showed more detailed view of the remnant cystic and common bile duct, which was folded up in the liver hilum

**Fig. 2:**
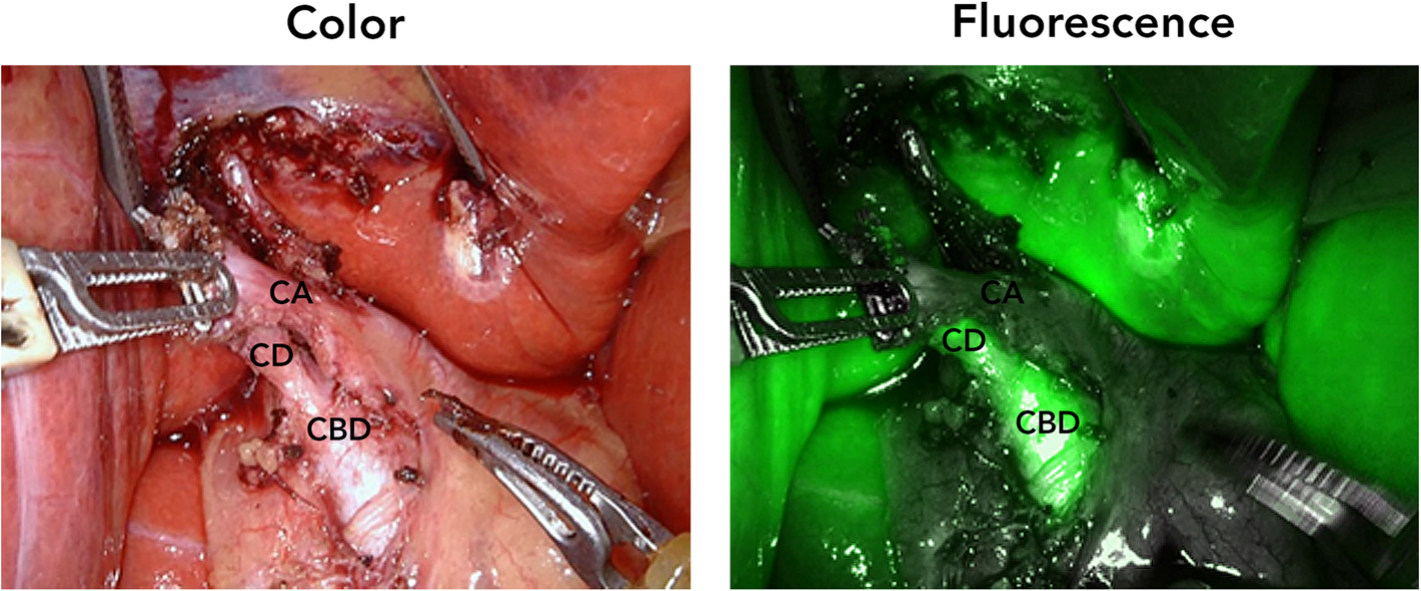
Color and fluorescence image of the remnant cystic duct and artery after dissection and detaching of the adhesions. *Abbreviations: CA: cystic artery; CD: (remnant) cystic duct; CBD: common bile duct*

**Fig. 3:**
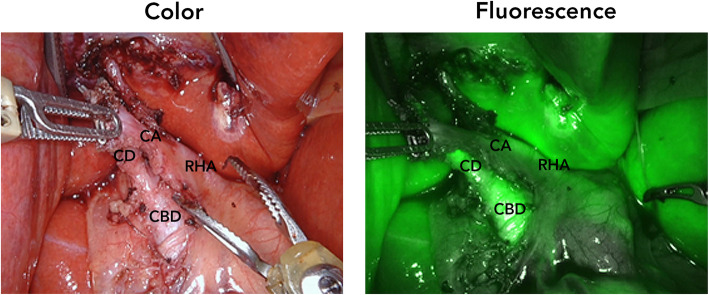
Confirmation of the arterial vasculature (remnant cystic artery and right hepatic artery) after injection of an extra bolus indocyanine green. *Abbreviations: CA: cystic artery; CD: (remnant) cystic duct; CBD: common bile duct; RHA: right hepatic artery*


After induction of general anaesthesia, 5 mg (2.5 mg/ml) of ICG was injected IV in order to visualize the biliary ducts. The patient was positioned in reverse Trendelenburg, and a small incision was made for introduction of 12 mm trocar at the umbilicus. Consequently, four additional robot trocars (8 mm) were placed under direct visualization. After docking, one experienced HPB surgeon and one surgical resident started the surgical procedure with exploration of the hepatoduodenal ligament until the liver hilum (Fig. 1). Using the *Da Vinci Xi (Intuitive Surgical Inc., Sunnyvale, USA)* fluorescence camera (Firefly™), the liver and CBD could be easily visualized, around 30 min. After IV injection of ICG. After adhesiolysis, the fluorescent remnant cystic duct and the non-fluorescent cystic artery were identified, also confirmed by the placed metal clips during the previous operation (Fig. 2). Using an additional IV injection of 2.5 mg ICG, the right hepatic artery and remnant cystic artery were confirmed with the Firefly™ (Fig. 3; Additional file 1). Resection of both the remnant cystic duct and artery was performed under fluorescence guidance. During resection of the cystic duct, some pus was relieved, indicating an infected state of the duct. The stump was controlled by double hemoclips. No gallstones were seen during surgery, which was confirmed ex vivo. Final pathology revealed presence of chronic and fibrosing inflammation in the remnant cystic duct. There were no complications during the procedure, which lasted 1 h and 35 min. The patient was checked postoperatively for jaundice, colic pain, and biochemically and none was present in the recovery period. The patient was discharged the same day and has not experienced similar symptoms as before during the first month of the follow-up.

**Additional file 1:** Injection of an extra bolus indocyanine green to confirm arterial vasculature. (MP4 78356 kb)


## Discussion and conclusions

A case report of a patient with a symptomatic remnant cystic duct, who underwent robot-assisted resection under fluorescence guidance, is presented. Good visualization of the remnant cystic duct and CBD was achieved by NIR fluorescence imaging using ICG, which helped to identify both structures and thereby resulting in an easier and safer surgical procedure. Intraoperative identification of a symptomatic cystic remnant duct can be challenging. Adhesions and inflammation could hamper visualization during surgery. As a consequence, BDI could occur, which is a complication with high mortality [9]. During surgery, no bile stones were visualized, although the resected specimen showed presence of chronic inflammation, which could indicate that a obstructing bile stone could have been located in the remnant cystic duct for a certain period of time.

Intraoperative assessment of the biliary ducts can be performed by different imaging techniques, of which IOC has been studied the most. However, the benefit of using IOC has not been proven in randomized clinical trials (RCTs), as level 1 evidence is low and all trials were insufficiently powered [10, 11]. Moreover, IOC is an invasive and time-consuming technique, and also involves the use of ionizing radiation [11, 12]. It could also cause BDI by itself, as insertion of a cannula for injection of contrast agents is necessary [13]. Compared to IOC, NIR fluorescence imaging of the biliary ducts using ICG does not prolong the operating time significantly, and could potentially decrease the operation time in difficult procedures, because of easy and fast detection of the biliary tract [14]. Currently, an ongoing international RCT is comparing the operation time of the procedure with and without the use of ICG fluorescence [15]. Furthermore, it is a non-hazardous and non-invasive technique, as it does not require use of radiation and cannot cause BDI [16]. Recently, a randomized controlled trial showed superiority of NIR fluorescence imaging with respect to standard white light conditions for detection of extrahepatic bile ducts during laparoscopic surgery [17]. Moreover, they showed encouraging results in their patient cohort of 639 patients (2 BDI in white light conditions vs. no BDI in de NIR fluorescence patient group) although due to low incidence of BDI, it is difficult to prove statistically significant differences between the two conditions. However, there are some limitations of this technique, such as the inability to detect bile stones and the limited penetration depth of NIR light up to 10 mm [5, 18].

Optimal injection times of ICG are still under discussion, although a systematic review showed that most studies used a preoperative administration, which was therefore recommended [18, 19]. We have shown, that injection of ICG after anesthesia, which was also the procedure in two recent RCTs, was also sufficient to visualize the biliary ducts during the whole procedure (30 min. – 125 min. Post injection) and could be easily implemented in clinical practice as the preparation phase of robotic surgery takes more time than conventional laparoscopy or laparotomy [15, 17]. As shown, the arterial vasculature could also be visualized by an extra IV injection of ICG, to confirm identification before resection.

In conclusion, this case demonstrates the use of NIR fluorescence imaging as a valuable tool for surgical guidance during a robot-assisted procedure of resection of the remnant cystic duct. This technique is safe and can easily be implemented in the surgical procedure. Therefore, we recommend to use ICG NIR fluorescence imaging during difficult robotic surgical procedures of the biliary tract, such as resection of a remnant cystic duct and gallbladder surgery in which inflamed surrounding tissue is expected. Nevertheless, a less invasive approach by using ERCP, remains the first line of treatment for patient with a symptomatic remnant cystic duct and therefore robotic surgery should only be performed in difficult cases.

## Data Availability

All patient data and clinical images adopted are contained in the medical files of the Leiden University Medical Center. The data supporting the conclusions of this article are included within the article and its figures.

## Abbreviations

BDI: Bile duct injury
CBD: Common bile duct
EUS: Endoscopic ultrasound
HPB: Hepatopancreaticobiliary
ICG: Indocyanine green
IOC: Intraoperative radiographic cholangiography
IV: Intravenous(ly)
MRCP: Magnetic resonance cholangiopancreaticography
NIR: Near-infrared
PCS: Post cholecystectomy syndrome
RCT: Randomized clinical trial

## References

[1] Kim JY, Kim KW, Ahn CS, Hwang S, Lee YJ, Shin YM, Lee MG (2008). Spectrum of biliary and nonbiliary complications after laparoscopic cholecystectomy: radiologic findings. AJR Am J Roentgenol.

[2] Pernice LM, Andreoli F (2009). Laparoscopic treatment of stone recurrence in a gallbladder remnant: report of an additional case and literature review. J Gastrointest Surg.

[3] Demetriades H, Pramateftakis MG, Kanellos I, Angelopoulos S, Mantzoros I, Betsis D (2008). Retained gallbladder remnant after laparoscopic cholecystectomy. J Laparoendosc Adv Surg Tech A.

[4] Walsh RM, Ponsky JL, Dumot J (2002). Retained gallbladder/cystic duct remnant calculi as a cause of postcholecystectomy pain. Surg Endosc.

[5] Vahrmeijer AL, Hutteman M, van der Vorst JR, van de Velde CJ, Frangioni JV (2013). Image-guided cancer surgery using near-infrared fluorescence. Nat Rev Clin Oncol.

[6] van Manen L, Handgraaf HJM, Diana M, Dijkstra J, Ishizawa T, Vahrmeijer AL, Mieog JSD (2018). A practical guide for the use of indocyanine green and methylene blue in fluorescence-guided abdominal surgery. J Surg Oncol.

[7] Agha RA, Borrelli MR, Farwana R, Koshy K, Fowler AJ, Orgill DP (2018). The SCARE 2018 statement: updating consensus surgical CAse REport (SCARE) guidelines. Int J Surg (London, England).

[8] Giljaca V, Gurusamy KS, Takwoingi Y, Higgie D, Poropat G, Stimac D, Davidson BR. Endoscopic ultrasound versus magnetic resonance cholangiopancreatography for common bile duct stones. Cochrane Database Syst Rev. 2015;26;(2):CD011549. 10.1002/14651858.CD011549 PMC6464848 25719224

[9] Halbert C, Altieri MS, Yang J, Meng Z, Chen H, Talamini M, Pryor A, Parikh P, Telem DA (2016). Long-term outcomes of patients with common bile duct injury following laparoscopic cholecystectomy. Surg Endosc.

[10] Buddingh KT, Nieuwenhuijs VB, van Buuren L, Hulscher JB, de Jong JS, van Dam GM (2011). Intraoperative assessment of biliary anatomy for prevention of bile duct injury: a review of current and future patient safety interventions. Surg Endosc.

[11] Ford JA, Soop M, Du J, Loveday BP, Rodgers M (2012). Systematic review of intraoperative cholangiography in cholecystectomy. Br J Surg.

[12] Buddingh KT, Weersma RK, Savenije RA, van Dam GM, Nieuwenhuijs VB (2011). Lower rate of major bile duct injury and increased intraoperative management of common bile duct stones after implementation of routine intraoperative cholangiography. J Am Coll Surg.

[13] Ishizawa T, Bandai Y, Ijichi M, Kaneko J, Hasegawa K, Kokudo N (2010). Fluorescent cholangiography illuminating the biliary tree during laparoscopic cholecystectomy. Br J Surg.

[14] Dip F, Roy M, Lo Menzo E, Simpfendorfer C, Szomstein S, Rosenthal RJ (2015). Routine use of fluorescent incisionless cholangiography as a new imaging modality during laparoscopic cholecystectomy. Surg Endosc.

[15] van den Bos J, Schols RM, Luyer MD, van Dam RM, Vahrmeijer AL, Meijerink WJ, Gobardhan PD, van Dam GM, Bouvy ND, Stassen LP (2016). Near-infrared fluorescence cholangiography assisted laparoscopic cholecystectomy versus conventional laparoscopic cholecystectomy (FALCON trial): study protocol for a multicentre randomised controlled trial. BMJ Open.

[16] Dip FD, Asbun D, Rosales-Velderrain A, Lo Menzo E, Simpfendorfer CH, Szomstein S, Rosenthal RJ (2014). Cost analysis and effectiveness comparing the routine use of intraoperative fluorescent cholangiography with fluoroscopic cholangiogram in patients undergoing laparoscopic cholecystectomy. Surg Endosc.

[17] Dip F, LoMenzo E, Sarotto L, Phillips E, Todeschini H, Nahmod M, Alle L, Schneider S, Kaja L, Boni L, et al. Randomized trial of near-infrared incisionless fluorescent cholangiography. Ann Surg. 2019 Jan 9. 10.1097/SLA.0000000000003178 30614881

[18] Boogerd LSF, Handgraaf HJM, Huurman VAL, Lam HD, Mieog JSD, van der Made WJ, van de Velde CJH, Vahrmeijer AL (2017). The best approach for laparoscopic fluorescence cholangiography: overview of the literature and optimization of dose and dosing time. Surg Innov.

[19] Vlek SL, van Dam DA, Rubinstein SM, de Lange-de Klerk ESM, Schoonmade LJ, Tuynman JB, Meijerink W, Ankersmit M (2017). Biliary tract visualization using near-infrared imaging with indocyanine green during laparoscopic cholecystectomy: results of a systematic review. Surg Endosc.

